# The Interleaving of Advertising Pages between Reading Matter

**Published:** 1890-01

**Authors:** 


### THE INTERLEAVING OF ADVERTISING PAGES BETWEEN READING MATTER.

It has become quite a custom of late among some of our contempories, to interleave their original and general reading matter with light-colored and fancy illustrated advertising pages.

We are strenuously opposed to this innovation, even though such a misplaced advertisement pays double for its usurped position.

The busy practitioner in scanning over his medical journal, is either bent on seeking information, or else is desirous of acquainting himself with the latest and best improvements in the matter of drugs, instruments and books. If he is seeking the latter, he certainly knows where he has to seek, or else he has recourse to the index; if he is intent on the former, he surely does not delight to have his train of thought suddenly collide with some highly artistic advertisement on 'beef peptonoids,' or other manufactured articles, no matter how valuable.

The truth is, the reader in subscribing for a journal, be it lay or professional, has acquired certain rights which the editor is bound to respect. He pays only for the reading matter, and expects to find that of the best quality, unadulterated, and in such a position where he is able to glean it with the least difficulty and least annoyance.

The advertiser also has his rights, and stipulates them in his contract. If his article be meritorious, and we believe such as appear in this journal are, he certainly gains nothing by attempting to force his goods upon the eye of the assiduous reader. In most cases, such pages are immediately destroyed, so as not to interfere with the continuation of some paper which otherwise would be disconnected.

The best journals, of whatever class, tongue, or clique, so arrange their matter that both reader and advertiser receive fullest remuneration. In such cases neither encroaches upon the other, both are distinct, and still both are brought into legitimate contact. Such is successful journalism.

---

We have recently received the thirty-seventh annual announcement of the Medical Department of the University of Vermont, located at Burlington in that State. Among other interesting features it contains a circular advertising "Lactated Food" and "Paine's Celery Compound," of the Wells, Richardson Company, also located at Burlington. We suppose this indicates some of the therapeutical measures used in this institution, or else the faculty would not have allowed the circular in their catalogue. We commend the enterprise of the Wells, Richardson Co., and also of the faculty of the college, though this is the first time to our knowledge that the annual announcement of a reputable medical school was ever used as an advertising medium for proprietary medicines. When we look over the list of names of the teachers printed in the catalogue we are at a loss to understand why this was allowed. Especially should the Dean of the Faculty have prevented it. We trust this example will not be followed by any other school. When medical colleges become the advertising agents of enterprising manufacturers of medicine, no matter how meritorious the article so advertised, they lower the standard of their usefulness and affront the dignity of the profession.

---

PHYSICIANS in possession of pathological specimens desirous of placing them in the museum of the Niagara University Medical College, will please send them to Wm. C. Krauss, M. D., 176 Franklin street, or to the college.

Such specimens will be properly prepared for exhibition with the name of the donor attached to the label.

